# The effects of the home-based exercise during COVID-19 school closure on the physical fitness of preschool children in China

**DOI:** 10.3389/fped.2022.932734

**Published:** 2022-08-30

**Authors:** Zhenwen Liang, Cheng Deng, Dan Li, Wai Leung Ambrose Lo, Qiuhua Yu, Zhuoming Chen

**Affiliations:** ^1^Department of Rehabilitation, The First Affiliated Hospital of Jinan University, Guangzhou, China; ^2^Department of Children’s Health Care, Zhongshan Torch Development Zone People’s Hospital, Zhongshan, China; ^3^Department of Rehabilitation Medicine, The First Affiliated Hospital, Sun Yat-sen University, Guangzhou, China

**Keywords:** children, home-based exercise, COVID-19, physical fitness, physical activity

## Abstract

**Background:**

Social distancing and school closures during the COVID-19 pandemic reduced the physical activities of the preschool children living in China. However, the effects of home-based exercise on the physical fitness of Chinese preschool children during COVID-19 school closures are still unknown. This study aimed to investigate the effects of home-based exercise on the physical fitness of Chinese preschool children during COVID-19 school closure.

**Methods:**

In this retrospective analysis, data from 1,608 Chinese preschool children (aged 3–5.5 years) in a second-tier city of Guangdong Province of China (Zhongshan city) were extracted from three successive National Physical Fitness Measurement (NPFM) from 2019 to 2021. NPFM consists of weight, height, and six subtests of physical fitness including 10-m shuttle run test (SRT), standing long jump (SLJ), balance beam walking (BBW), sit-and-reach (SR), tennis throwing (TT), and double-leg timed hop (DTH) tests. The change differences or change ratios of all the items in NPFM between any two successive years from 2019 to 2021 were compared. The exercise profiles about home-based and outdoor exercise before, during, and after COVID-19 school closure were obtained from 185 preschool children *via* retrospective telephone survey.

**Results:**

Between 2019 and 2021, 1,608 preschool children were included in this study. We observed larger changes in SLJ, SR, TT, and DTH tests during school closure than after school closure. But the children showed lower reduction rates in the completion time of SRT and BBW. During school closure, higher change ratios in SLJ and TT were observed in the children primarily participating in home-based exercise than those primarily participating in outdoor exercise. However, no statistical differences were observed in the changes in SRT and BBW between home-based and outdoor training groups.

**Conclusion:**

The home-based exercise program might be an alternative approach to improve the physical fitness of preschool children during COVID-19 school closure, but could not be beneficial to speed-agility and balance functions. A specific guideline geared toward a home-based exercise program during the COVID-19 outbreak is highly needed.

## Introduction

The emergence of the severe acute respiratory syndrome caused by coronavirus 2 (SARS-CoV-2) (COVID-19) has rapidly become a massive global health crisis. The aggressiveness and the speed of propagation of the virus contributed to the pandemic in China. To prevent the spread of COVID-19, human movement and physical interactions were restricted all over China to promote social distancing (1), especially in China’s first- and second-tier cities due to the high population density with a higher risk of spread of COVID-19 (2). All of the kindergartens in China and many other countries were closed and preschool children mainly stayed at home without the regular practice of physical activity in their daily life (3–5). It was reported that the children in China and other countries spent less time on physical activity and engaged in more sedentary behavior during the early-COVID-19 period as compared to before the pandemic (5, 6). Gatti et al. found that most of the adolescent participants were less active but reported adequate physical fitness levels during the COVID-19 pandemic (7). This misperception of reported physical activity and physical fitness could reduce the future physical activity level, which should lead to attention to physical activity promotion during the COVID-19 pandemic. Lots of evidence reported that a decreased amount of physical activities or sedentary behavior may subsequently affect physical fitness including muscular strength, agility, flexibility, cardiorespiratory endurance, and body composition (8–10).

Physical fitness is a fundamental factor and potential biomarker for body development, cognition, and academic achievement for children (11, 12). Age 0–6 years is the fastest and most important stage of body growth and development. It is essential to retain the physical fitness of preschoolers during COVID-19 school closure. Therefore, exercise programs and policy strategies should be geared toward increasing the physical activities of children (5, 6, 13). According to the notice promoted by the National Health Commission of the People’s Republic of China (14) and the World Health Organization guidelines on physical activity and sedentary behavior (15), children and adolescents were recommended to have at least 60 min of light-to-moderate physical activity per day to improve physical fitness during the pandemic. Home-based exercise was recommended by the General Administration of Sport of China to reduce physical inactivity and sedentary behavior during the COVID-19 pandemic (16, 17). Home-based exercise is not a novel topic. Its positive effects on physical and psychological health have been reported in clinical populations (18, 19). During COVID-19 school closure, children living in the US were more likely to perform physical activities at home to reduce sedentary behavior (6, 20). A study conducted by Pu et al. reported that the Chinese people staying at home preferred dancing and yoga, walking or jogging, stretching exercises (e.g., leg lift, etc.), housework (e.g., cooking, cleaning, etc.), and other activities (e.g., rope skipping, weight lifting, Tai chi, etc.) during the COVID-19 outbreak (20). Home-based exercise seems to have a positive effect on the lifestyle and physical fitness of the children during school closure (5). However, there was currently no specific guidelines addressing the type and amount recommended for home-based exercise. Therefore, the effects of home-based exercise on different attributes of physical fitness of Chinese preschool children during COVID-19 school closure are unknown.

The present study aimed to investigate the effects of home-based exercise on different attributes of physical fitness of Chinese preschool children during COVID-19 school closure. It was hypothesized that home-based exercise during the COVID-19 school closure period had positive effects on the physical fitness of preschool children.

## Materials and methods

### Study design and participants

This is an observational retrospective study. Physical fitness data from 2019 to 2021 of preschool children aged 3–5.5 years were extracted from the annual physical examination in 2019, 2020, and 2021 conducted by Zhongshan Torch Development Zone People’s Hospital of Guangdong Province in China by using the protocol of the preschool children version of National Physical Fitness Measurement (NPFM). During COVID-19, the government policy of school closure to enhance social distancing was strictly carried out in China’s first- and second-tier cities (1), which had higher population density likely leading to a higher speed of propagation of COVID-19 (2, 21). Therefore, the Chinese preschool children in one representative of China’s second-tier cities (Zhongshan City, Guangdong) were recruited in the present study. The population density of Zhongshan City is near to that of Guangzhou City. The sample recruitment in this study used stratified sampling, which was that around 600 children were randomly selected from each of the 3-year groups of 10 kindergartens in Zhongshan City. The exclusion criteria were as follows: (1) participants with missing data; and (2) participants with biologically implausible values (defined by the logical check boundary value for the NPFM test items). Ethical approval was obtained from the ethical committee of the First Affiliated Hospital of Jinan University (ETHICS No.KY-2021-093). Informed consent was deemed not required by the ethics committee due to the retrospective nature of the study.

### Study settings and instrumentation

The physical fitness of the preschool children in this study was assessed by using NPFM, which consisted of a battery of comprehensive physical fitness tests designed by the China General Administration of Sport in 2000 (22). NPFM includes eight assessment items, which are weight, height, and six physical fitness subtests. Six physical fitness subtests included 10-m shuttle run test (SRT), standing long jump (SLJ), balance beam walking (BBW), sit-and-reach (SR), tennis throwing (TT), and double-leg timed hop (DTH) tests (23). It was reported that NPFM had acceptable reliability and sensitivity to assess the physical fitness of preschool children in China (22). The details of the NPFM test were described as follows.

#### 10-m shuttle run test

In the 10-m SRT, each participant was required to run at full speed, touch the target object at a distance of 10 m from the starting line, and then run back to the starting line as quickly as possible. The completion time for this test was recorded.

#### Standing long jump test

In the SLJ test, each participant stood behind the starting line and jumped as far as possible with arms swinging and landing with both feet. The distance from the starting line to the heel of the rear landing foot was recorded.

#### Tennis throwing test

During the TT test, each participant was required to stand behind the starting line and throw a tennis ball forward as far as possible. If the foot of the participant stepped on or over the starting line during or after throwing, the participants were asked to redo the test again. The distance from the starting line to the first landing point of the ball was recorded.

#### Double-leg timed hop test

Ten rectangular soft blocks (10 cm [length] × 5 cm [width] × 5 cm [height]) were placed 50 cm apart from each other. Before the DTH test, each participant was required to stand with their feet together at 20 cm behind the first block. Each participant was asked to jump over all the barriers on both legs as fast as possible after the start signal. Participants were required to redo the test if they stepped or kicked on the block during the test. The time to complete the DTH test was recorded.

#### Sit-and-reach test

Before the test, the participant sat on the ground with bare feet together and knees straight. The soles of their feet should press against the edge of the sit-and-reach box, which was regarded as zero point. The participants were required to bend their trunks forward and push the moveable marker of the scale plate with their fingertips as far as possible without bending their knees. The distance from the starting point to the place where the marker stopped was recorded. Trials with a stopped marker that failed to pass the zero point were recorded as negative values.

#### Balance beam walking test

The participant was required to walk along a 3 m-long, 10 cm wide, and 30 cm high balance beam as fast as possible. During the test, the participant was required to keep the arm at a 90^°^ abduction position. If the participant fell from the beam during the test, the participant was required to redo the test. The completion time was recorded.

### Procedure

NPFM was conducted by the trained staff of the Zhongshan Torch Development Zone People’s Hospital in Zhongshan City of Guangdong Province on December 2019, October 2020, and July 2021. At the beginning of the NPFM test, the body weight and height of each participant were measured. Then, each participant performed six subtests involved in the NPFM in randomized order. According to the guideline of NPFM, no previous familiarization session was given. After providing verbal instruction and demonstration, each participant performed each test two times. Each subtest of NPFM was conducted by two fixed staff at the same location of each kindergarten across 3 years. This further enhances the reliability of the data set. The data of the six subtests were retrieved from the database from the information system of Zhongshan Torch Development Zone People’s Hospital for data analysis. The exercise profiles of 185 children were randomly selected from 1,608 participants before (before December 2019), during (from Jan to September 2020), and after school closure (after September 2020). Exercise profiles were obtained from the children’s caregivers *via* telephone survey retrospectively on December 2021. The exercise profile included the primary exercise area, daily exercise duration, the time spent in indoor exercise per week, the frequency of outdoor exercise per week, and the time spent in outdoor exercise per time. All the questions about the exercise profile in the telephone survey were shown in Table 1. The exercise profiles of the first 10 children were obtained again *via* telephone after 1 week of the first telephone survey for assessing the test-retest reliability of the questions about exercise profiles. The test-retest reliability using Cronbach’s Alpha was high (range from 0.899 to 1). The sample size for each age level was 24 for 3.0 years, 40 for 3.5 years, 41 for 4.0 years, 44 for 4.5 years, and 36 for 5.0 years.

**TABLE 1:** The questions about the exercise profile in the telephone survey.

1.	What was your primary mode of exercise? Outdoor or home-based exercise before/during/after the school closure in 2020?
2.	How much time did you spend on daily exercise before/during/after the school closure in 2020?
3.	How much time did you spend on indoor exercise per week before/during/after the school closure in 2020?
4.	How often did you do outdoor exercise in a week before/during/after the school closure in 2020?
5.	How much time did you spend on outdoor exercise per week before/during/after the school closure in 2020?

### Data analysis

Because the outcome measured in the SR test was the distance from the start point to the place where the marker stopped, the scores of some participants were zero. Thus, the change difference of distance in the SR test and the change ratios of other NPFM outcomes were used in the data analysis. The outcome variables of the present study included the change differences in weight and height and the change differences/ratios of the NPFM outcomes between 2019 and 2020 (during school closure) and between 2020 and 2021 (after school closure) among different age groups. All the outcome variables were analyzed by two-way repeated measures ANOVA: the within-subject factor were time (between 2019 and 2020 or between 2020 and 2021); the between factor was the age group (3, 3.5, 4, 4.5, or 5 years). The Greenhouse–Geisser correction was used when Mauchly’s test of sphericity was violated. *Post hoc* pairwise comparisons were conducted when a significant time × age interaction effect was observed. The Chi-square test was performed to compare the exercise profile of the participants among different periods, which involved before, during, and after COVID-19 school closure. The change differences or change ratios of age and all the outcome variables between the children primarily participating in outdoor exercise and those participating in home-based exercise were compared by independent-sample *t*-test. The significance level was set at *p* < 0.050.

## Results

### Demographic characteristics

From 2019 to 2021, 1,608 preschool children (boys: *n* = 867, girls: *n* = 741) in the kindergarten participated in the annual NPFM. The demographic characteristics of all the participants are shown in Table 2.

**TABLE 2:** The demographic characteristics of all the participants.

		**Age**				
		3.0	3.5	4.0	4.5	5.0
Gender	Boy/Girl	123/100	183/151	177/179	241/209	143/102
Weight	Mean (*SD*)	15.30 (1.77)	16.17 (1.72)	17.10 (2.15)	18.06 (2.20)	19.15 (2.97)
Height	Mean (*SD*)	98.73 (3.65)	101.93 (3.81)	105.46 (4.49)	108.79 (4.26)	111.66 (4.26)

SD, denotes standard deviation.

### The change differences of weight and height

**FIGURE 1:**
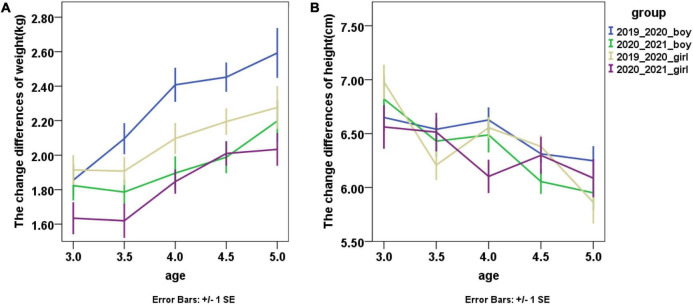
The change differences of weight and height between 2020 and 2019 and between 2021 and 2020, **(A)** for weight; **(B)** for height.

The change differences of weight and height between 2019 and 2020 and between 2020 and 2021 among different age groups are shown in Figure 1. For both boys and girls, the time effect was significant in the change differences of weight [boys: *F*(1, 862) = 30.98, *p* < 0.001; girls: *F*(1, 736) = 19.41, *p* < 0.001], but not significant in the change difference of height [boys: *F*(1, 862) = 1.57, *p* = 0.210; girls: *F*(1, 736) = 0.49, *p* = 0.486]. The age effect was also significant in both weight [boys: *F*(4, 862) = 7.98, *p* < 0.001; girls: *F*(4, 736) = 7.29, *p* < 0.001] and height [boys: *F*(4, 862) = 10.50, *p* < 0.001; girls: *F*(4, 736) = 6.95, *p* < 0.001]. The time × age interaction effects and height were not significant in the change differences of weight [boys: *F*(4, 862) = 1.65, *p* = 0.160; girls: *F*(4, 736) = 0.13, *p* = 0.970] and height [boys: *F*(4, 862) = 0.57, *p* = 0.688; girls: *F*(4, 736) = 1.71, *p* = 0.146]. These findings suggested that both boys and girls had larger changes in weight during COVID-19 school closure.

### The change ratios/difference of the outcomes of six subtests in national physical fitness measurement

**FIGURE 2:**
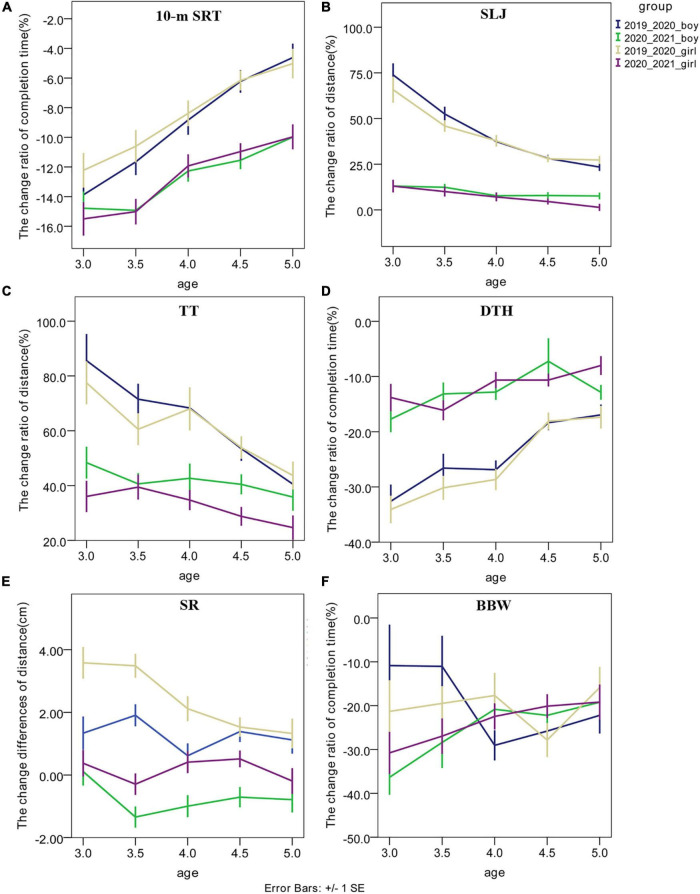
The change ratios or change difference in the six subtests of NPFM between 2020 and 2019 and between 2021 and 2020. **(A)** for 10-m SRT; **(B)** for SLJ test; **(C)** for TT test; **(D)** for DTH test; **(E)** for SR test; **(F)** for BBW test. SRT denotes 10-m shuttle run, SLJ denotes standing long jump, TT denotes tennis throwing, DTH denotes double-leg timed hop, SR denotes sit-and-reach, and BBW denotes balance beam walking.

The change ratios of completion time in the 10-m SRT between 2020 and 2019 and between 2021 and 2020 were shown in Figure 2A. In the 10-m SRT, the time (*p* < 0.001) and age (*p* < 0.001) effects were significant in the change ratios of completion time of the 10-m SRT test. The time × age interaction effects were not significant for boys and girls (*p* > 0.050). All the within-subject and between-subject effects of all NPFM outcome variables were shown in Table 3. These findings suggested that both boys and girls had lower growth rates in the capacity of speed-agility during the school closure period of the COVID-19 pandemic.

**TABLE 3:** All the within-subject and between-subject effects of all NPFM outcome variables.

		df		*F*		*p*-values	
		Boy	Girl	Boy	Girl	Boy	Girl
SRT	Time	1,862	1,736	29.61	37.45	<0.001	<0.001
	Age	4,862	4,736	40.28	28.91	<0.001	<0.001
	Time × Age	4,862	4,736	1.35	0.24	0.250	0.919
SLJ	Time	1,862	1,736	266.35	224.38	<0.001	<0.001
	Age	4,862	4,736	42.68	30.26	<0.001	<0.001
	Time × Age	4,862	4,736	13.50	4.71	<0.001	0.001
TT	Time	1,862	1,736	30.55	47.745	<0.001	<0.001
	Age	4,862	4,736	9.59	6.45	<0.001	<0.001
	Time × Age	4,862	4,736	2.01	0.90	0.092	0.464
DTH	TIME	1,862	1,736	40.54	97.10	<0.001	<0.001
	Age	4,862	4,736	8.52	21.24	<0.001	<0.001
	Time × Age	4,862	4,736	1.04	3.30	0.385	0.011
SR	Time	1,862	1,736	47.82	63.12	<0.001	<0.001
	Age	4,862	4,736	2.09	4.51	0.080	0.001
	Time × Age	4,862	4,736	1.37	3.94	0.243	0.004
BBW	Time	1,862	1,736	2.53	1.13	0.112	0.289
	Age	4,862	4,736	0.58	1.51	0.680	0.197
	Time × Age	4,862	4,736	3.34	1.09	0.010	0.362

df, degree of freedom; SRT, 10-m shuttle run test; SLJ, standing long jump; TT, tennis throwing; DTH, double-leg timed hop; SR, sit-and-reach; BBW, balance beam walking.

The change ratios of distance in the SLJ between 2020 and 2019 and between 2021 and 2020 are shown in Figure 2B. In the SLJ, the time (*p* < 0.001) and age (*p* < 0.001) effects were significant in the change ratios of distance. The time × age interaction effects were significant for boys (*p* < 0.001) and girls (*p* = 0.001). The *post hoc* analyses of the interaction effect showed that boys and girls of all the age groups exhibited higher change ratios of distance in the SLJ between 2020 and 2019 than those between 2021 and 2020 (*p* < 0.001). These findings suggested that preschool children had larger growth rates in muscle power of lower limb during the COVID-19 school closure period.

The change ratios of distance in TT between 2020 and 2019 and between 2021 and 2020 were shown in Figure 2C. In TT, the time (*p* < 0.001) and age (*p* < 0.001) effects were significant in the change ratios of distance. The time × age interaction effects were not significant (*p* > 0.050). These findings suggested that preschool children had larger growth rates in muscle strength of the upper limb during the COVID-19 school closure period.

The change ratios of completion time in the DTH between 2020 and 2019 and between 2021 and 2020 were shown in Figure 2D. In the DTH test, the time (*p* < 0.001) and age (*p* < 0.001) effects were significant in the change ratios of completion time. The time × age interaction effect was significant in girls (*p* = 0.011) but not significant in boys (*p* = 0.385). The *post hoc* analyses of this significant interaction effect showed that the girls of all the age groups exhibited a larger reduction in the change ratios of completion time in the DTH test between 2020 and 2019 than those between 2021 and 2020 (*p* < 0.010). These findings suggested that both preschool girls and boys had larger growth rates in motor coordination and muscle strength of low limb during COVID-19 school closure.

The change differences of distance in the SR test between 2020 and 2019 and between 2021 and 2020 were shown in Figure 2E. In the SR test, the time effects were significant in the change differences of distance in SR test (*p* < 0.001). The age effect and time × age interaction effect were significant in girls (*p* < 0.050) but not significant in boys (*p* > 0.050). The *post hoc* analyses of the significant interaction effect showed that girls of all the age groups exhibited larger change differences of distance in the SR test between 2020 and 2019 than those between 2021 and 2020 (*p* < 0.010). These findings suggested that both preschool girls and boys had larger growth rates in body flexibility during the COVID-19 school closure period.

The change ratios of completion time in the BBW between 2020 and 2019 and between 2021 and 2020 were shown in Figure 2F. In the BBW test, the time and the age effects were not significant in the change ratios of completion time (*p* > 0.050). The time × age interaction effect was significant in boys (*p* = 0.010) but not significant in girls (*p* > 0.050). The *post hoc* analyses of the interaction effect showed that boys aged 3.0 and 3.5 years exhibited more reduction ratios of completion time of boys aged 3.0 and 3.5 years in the BBW test between 2021 and 2020 than those between 2020 and 2019 (*p* < 0.050). These findings suggested that preschool boys at younger age had lower growth rates in the balance function of the BBW during the school closure period.

### The exercise profiles of preschool children before, during, and after school closure

**FIGURE 3:**
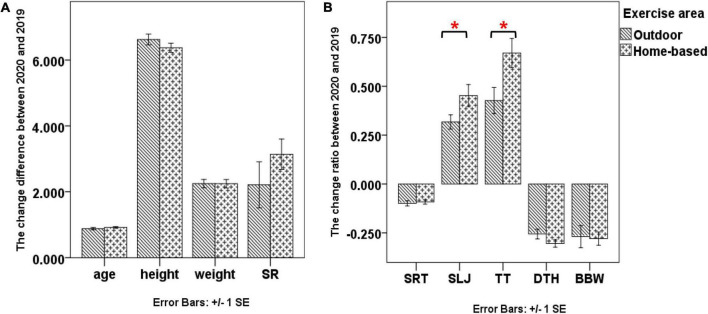
The changes of demographic characteristics and all the NPFM outcomes between 2020 and 2019 for the children primarily participating in outdoor exercise and those participating in home-based exercise. **(A)** for the change differences of age, height, weight, and SR test; **(B)** for the change ratios of SRT, SLJ, TT, DTH, and BBW tests. SR, sit-and-reach; SRT, 10-m shuttle run test; SLJ, standing long jump; TT, tennis throwing; DTH, double-leg timed hop; BBW, balance beam walking. *Denotes *p* < 0.050.

The exercise profiles of the 185 preschool children before, during, and after school closure are shown in Table 4. The majority of children had exercised outdoor before (83.2%) and after (88.1%) COVID-19 school closure, compared to during COVID-19 school closure (33.5%), whereas the majority of children preferred home-based exercise during school closure (66.5%). During school closure, the proportions of the children who performed indoor exercise per week for 2–4 h (41.1%) and 6–8 h (17.3%) were statistically higher than those before (2∼4 h:24.9%; 6∼8 h:8.6%) and after school closure (2∼4 h:27.0%; 6∼8 h:6.5%). The proportion of children without outdoor exercise was 14.1% during the school closure period, which was highly larger than those before (1.6%) and after (1.6%) COVID-19 school closure. The change ratios in SLJ (*t* = −1.994, *p* = 0.048, 95% Confidence Interval (CI):−0.267 to −0.001) and TT (*t* = −2.416, *p* = 0.017, 95% CI:−0.441 to −0.044) between 2020 and 2019 were higher in the children primarily participated in home-based exercise than those primarily participated in outdoor exercise (Figure 3B). The change differences of age, weight height and the changes of the other NPFM outcomes between 2020 and 2019 were not statistically significant (Figure 3). However, there seemed to be a larger change difference of SR distance and a larger reduction in the change ratios of DTH completion time during school closure in the home-based group than in the outdoor group.

**TABLE 4:** The exercise profile of 185 children before, during, and after COVID-19 school closure.

		Time			χ^2^	*P*
		Before school closure	During school closure	After school closure		
The primary exercise area	Outdoor	154 (83.2%)	62 (33.5%)	163 (88.1%)	155.97	<0.001
	Home-based exercise	31 (16.8%)	123 (66.5%)	22 (11.9%)	155.97	<0.001
Daily exercise duration	<0.5 h	23 (12.4%)	67 (36.2%)	23 (12.4%)	43.03	<0.001
	0.5∼1 h	76 (41.1%)	74 (40.0%)	77 (41.6%)	0.11	0.949
	> 1 h	86 (46.5%)	44 (23.8%)	85 (45.9%)	26.16	<0.001
The time spent in indoor exercise per week	<2 h	58 (31.4%)	27 (14.6%)	57 (30.8%)	17.62	<0.001
	2∼4 h	46 (24.9%)	76 (41.1%)	50 (27.0%)	13.41	0.01
	4∼6 h	47 (25.4%)	31 (16.8%)	46 (24.9%)	5.01	0.075
	6∼8 h	16 (8.6%)	32 (17.3%)	12 (6.5%)	12.56	0.002
	>8 h	18 (9.7%)	19 (10.3%)	20 (10/8%)	0.12	0.943
The frequency of outdoor exercise per week	0 time	3 (1.6%)	26 (14.1%)	5 (2.7%)	30.52	<0.001
	1∼2 times	59 (31.9%)	96 (51.9%)	58 (31.4%)	21.44	<0.001
	3∼4 times	65 (35.1%)	42 (22.7%)	67 (36.2%)	9.70	0.007
	≥5 times	58 (31.4%)	21 (11.4%)	55 (29.7%)	24.93	<0.001
The time spent in outdoor exercise per time	0 h	3 (1.6%)	26 (14.1%)	3 (1.6%)	35.09	<0.001
	<0.5 h	33 (17.8%)	74 (40.0%)	28 (15.1%)	37.41	<0.001
	0.5∼1 h	67 (36.2%)	46 (24.9%)	74 (40.0%)	10.28	0.006
	>1 h	82 (44.3%)	39 (21.1%)	80 (43.2%)	27.57	<0.001

## Discussion

The present study investigated the effects of home-based exercise on different types of physical fitness of Chinese preschool children during COVID-19 school closure. The results showed an increase in weight and larger changes ratios in SLJ, SR, TT, and DTH tests during COVID-19 school closure. The reduction rate in the completion time of SRT and BBW was lower during the school closure period than before school closure. Larger change ratios was noted in SLJ and TT tests in the children who primarily participated in home-based exercise than those who primarily participated in outdoor exercise. These findings suggested that preschool children had larger growth rates in muscle strength, muscle power, muscle coordination, and body flexibility, which was likely related to the home-based exercise during COVID-19 school closure. The enhancement in muscle strength, muscle power, muscle coordination, and body flexibility related to the home-based exercise during COVID-19 school closure supported our hypothesis. However, no statistical differences were observed in the change ratios of the completion time in SRT and BBW tests during the school closure period between home-based and outdoor training groups. The findings in SRT and BBW tests suggested that the growth rates of speed-agility and balance functions were decreased for the preschool children during COVID-19 school closure, which were new findings and were inconsistent with our hypothesis.

This study found that preschool children had larger growth rates in muscle strength, muscle power, muscle coordination, and body flexibility during COVID-19 school closure. Larger growth rates in muscle strength, muscle power, muscle coordination, and body flexibility in the present study may result from the home-based exercise, which was supported by those reported in the previous studies (24–27). A meta-analysis conducted by Chaabene et al. (24) showed that home-based exercise during the COVID-19 pandemic appeared to be effective to improve muscle strength, muscle power, and body flexibility for elderly people (24). Some recent studies also found that a home-based exercise program was beneficial for physical fitness during the COVID-19 pandemic due to the requirement of social distancing (24, 26). The football players aged 12–17 years were required to practice at home to maintain their body composition, cardiorespiratory, and muscular fitness during the COVID-19 pandemic (27). Around a half of Singapore students with lower age levels primarily did aerobic activities (e.g., running) as home-based physical activity during COVID-19 (25). Aerobic training was reported to be able to enhance leg strength and power (28). In the present study, the possible reason for the improvement in muscle strength, muscle power, and muscle coordination during COVID-19 school closure was related to the implementation of a home-based exercise program as proposed by the Government of China (14, 29). The National Health Commission of China emphasized the need for children and adolescents to engage in at least 1 h of daily physical activity in school (14). To cope with the policies of restricted movements due to COVID-19, home-based exercise was recommended as an alternative approach to increase physical activity and improve physical fitness by the Government of China (16, 17). In the early phase of the COVID-19 pandemic, the most frequently reported physical activities were free play/unstructured physical activities at home (e.g., running around, roller skating, and biking). These types of exercise are beneficial for muscle strength, muscle power, and muscle coordination (6). The recommended home-based exercise policies and the exercise preference of preschool children were beneficial to increasing the growth rates of muscle strength, muscle power, muscle coordination, and body flexibility for preschoolers.

Our findings about the exercise profile before, during, and after the COVID-19 school closure of the 185 children suggested that most of the preschool children preferred home-based exercise during the school closure period. Higher change ratios in SLJ and TT and an increasing trend in the change ratios in SR and DTH were observed in the children who primarily participated in home-based exercise than those who primarily participated in outdoor exercise during school closure. These findings suggested that home-based exercise during school closure could be associated with muscle strength, muscle power, muscle coordination, and body flexibility, which further explained the reason for our findings that the larger growth rates in muscle strength, muscle power, muscle coordination, and body flexibility were observed during COVID-19 school closure than after COVID-19 school closure. These findings were consistent with the previous studies, which reported that home-based exercise programs could enhance physical fitness during COVID-19 school closure (24, 26). However, the primary types of home-based exercise were muscle-strengthening activities and aerobic activities (25). Only a small portion of Singapore’s children participated in ball games or other forms of exercise that required speed-agility and balance (25). Therefore, in this study, the growth rates of speed-agility and balance functions were decreased and no between-group difference (outdoor vs. home-based) was found in the growth rates of speed-agility and balance functions during COVID-19 school closure.

Speed relates to the ability to perform a movement within a short period, while agility relates to the ability to rapidly and accurately change the position/direction of the entire body in response to a stimulus. Speed-agility are mostly associated with enhanced performance in sports and motor skills for children (30). The present study found that the growth rate of speed-agility function was decreased for pre-school children during COVID-19 school closure, which was a new finding. The potential reason was that the amount of training on speed-agility capacity was insufficient during the school closure period. This was supported by the home-based exercise program proposed by the Government of China, which only included one item related to speed-agility training (29). In addition, the available space for home-based exercise is very limited which reduces the training options for enhancing speed-agility (31). Therefore, the growth rate of the speed-agility function decreased during the COVID-19 school closure.

In the present study, the growth rate of balance function was decreased during COVID-19 school closure only for boys of younger age. There were no change differences in other age groups or in girls. These findings were another new findings, but inconsistent with those reported in the previous studies (24, 32). Chaabene et al. (24) indicated that home-based exercise during the COVID-19 pandemic appeared effective to improve balance for elderly people. Another study found that 16-week of home-based strength exercises improved balance function in adults older than 50 years (32). However, there is insufficient data on the effects of home-based exercise available in the preschooler population for direct comparison. Therefore, the differences in the effects of the home-based exercise on the balance function between the present study and the previous studies might be related to the different sample populations. The sample population in the present study was preschool children, whereas those in the previous studies were adults or elderly people. The home-based exercise program proposed by the Government of China, also recommended the exercises to improve balance for the children (29). Preschool children, especially the boys of the lower age group have poor awareness of danger (33). It was reported that boys aged 2–4 years exposed to exercise equipment at home were most at risk for exercise equipment-related injury (33). The exercises that have a high risk of injury to children such as walking on a balance beam are required to be performed under parents’ supervision (34). The home-based exercises that are at low risk of injury, such as walking, running, and jumping on the floor, are preferred during the COVID-19 period (34). Another reason related to home-based exercises that is not beneficial for children’s balance function is likely related to the requirement of specific equipment for balance training. This may not always be possible due to the limited space available at home. Thus, it was not easy for the children to perform the balancing exercise that requires large space and specific equipment such as a balance beam (31). The previous study also found that adolescent football players need to focus on balance training to prevent injury in the restart period after the COVID-19 pandemic because home-based training during the COVID-19 pandemic was insufficient for the football players (27). The potential reason for the changes of speed-agility and balance functions between during and after school closure was further supported by no significant differences in SRT and BBW between home-based and outdoor groups, which suggested that the current home-based exercise during school closure could not effectively improve the speed-agility and balance performance of children. Therefore, specific home-based exercises tapping on speed-agility and balance functions should be recommended to the preschool children in the further national policy.

This study also found that both boys and girls had larger growth changes in weight during COVID-19 school closure. These findings were consistent with the previous studies that reported larger changes in weight resulting from reduced physical activities during the COVID-19 pandemic (3, 35–37). A body of evidence reported the unhealthy weight gain during the summer holidays when schools are closed, rather than during the school year (38, 39). Von Hippel and Workman reported that the prevalence of childhood obesity and overweight decreased across 3 school years but increased during the summer recess (38). The changes of diet plan and physical activity levels due to the kindergarten closures during the COVID-19 outbreak mainly contributed to the increased growth changes in weight (3).

The strengths of the present study included its comprehensive assessment of physical fitness with a relatively large regional-representative sample size and comparison across a 3-year period, which could be beneficial to make a relatively concrete conclusion, and confirm the robustness of the present data and support the original hypothesis. The present study also has several limitations. First, the retrospective survey about exercise profiles was completed by only a small portion of preschool children recruited in this study, which might lead to bias due to the memory recall error. Thus, it could not be ascertained that the recalled exercise profile accurately represents the actual amount of exercise time of the sample population. Second, we only collected data from one city data in Southern China. The findings of the present study could not be generalized to other regions of China, particularly in the regions that have different resource levels than Southern China. Future studies may consider more data from other regions of China. Finally, the data about the types of physical activities and family environments were not collected, which might substantially affect the children’s physical exercise profile and subsequently have an impact on physical fitness (40). Future studies should consider these factors to have a better understanding of the effects of home-based exercise on physical fitness.

## Conclusion

These findings suggested that home-based exercise program might be an alternative approach to improve physical fitness during COVID-19 school closure. But the specific home-based exercises for enhancing the speed-agility and balance functions of preschool children should be put more attention to in the further national policy. A specific guideline for the home-based exercise program during COVID-19 school closure is highly needed.

## Data availability statement

The raw data supporting the conclusions of this article will be made available by the authors, without undue reservation.

## Ethics statement

The studies involving human participants were reviewed and approved by the Ethical Committee of the First Affiliated Hospital of Jinan University. Written informed consent from the participants’ legal guardian/next of kin was not required to participate in this study in accordance with the national legislation and the institutional requirements.

## Author contributions

ZL and CD designed and conceptualized the study, and completed the data collection, analysis, and interpretation. DL completed the data analysis and interpretation. WL and QY participated in the data analysis and drafting of the manuscript. QY and ZC revised the manuscript, interpreted the data, and managed the trial. All authors reviewed the manuscript.
